# Who is going to turn on the ventilators?

**DOI:** 10.31744/einstein_journal/2021AO6211

**Published:** 2021-10-13

**Authors:** Marcelo Cunio Machado Fonseca, Gabriela Tannus Branco de Araújo, Fulvio Alexandre Scorza, Paulo Sérgio Lucas da Silva, Teresa Raquel de Moraes Andrade, Daniela Farah, Dayan Sansone

**Affiliations:** 1 Escola Paulista de Medicina Universidade Federal de São Paulo São PauloSP Brazil Escola Paulista de Medicina, Universidade Federal de São Paulo, São Paulo, SP, Brazil.; 2 Hospital do Servidor Público Municipal de São Paulo São PauloSP Brazil Hospital do Servidor Público Municipal de São Paulo, São Paulo, SP, Brazil.

**Keywords:** COVID-19, Coronavirus infections, SARS-CoV-2, Health personnel, Intensive care units

## Abstract

**Objective:**

To analyze the COVID-19 pandemic in Brazil, a continental-sized country, considered as an emerging economy but with several regional nuances, focusing on the availability of human resources, especially for intensive care units.

**Methods:**

The database of the National Registry of Health Facilities was accessed. Healthcare professionals in the care of COVID-19 were georeferenced. We correlated the number of professionals with the parameters used by the World Health Organization. According to the Brazilian Intensive Care Medicine Association, we correlated the data for adult intensive care unit beds in each state with the number of professionals for each ten intensive care unit beds. The number of professionals, beds, and cases were then organized by state.

**Results:**

The number of physicians per 100 thousand inhabitants followed the World Health Organization recommendations; however, the number of nurses did not. The number of intensivists, registered nurses, nurse technicians specialized in intensive care, and respiratory therapists, necessary for every ten intensive care beds, was not enough for any of these professional categories. A complete team of critical care specialists was available for 10% of intensive care unit beds in Brazil.

**Conclusion:**

There is a shortage of professionals for intensive care unit, as we demonstrated for Brazil. Intensive care physical resources to be efficiently used require extremely specialized human resources; therefore, planning human resources is just as crucial as planning physical and structural resources.

## INTRODUCTION

The coronavirus disease 2019 (COVID-19) is a viral respiratory disease caused by severe acute respiratory syndrome coronavirus 2 (SARS-Cov-2), which was first detected, in Wuhan, China, in December 2019. Patients may be classified as asymptomatic or symptomatic, and the clinical presentation comprises a broad range of unspecified symptoms, such as fever, dry cough, dyspnoea, headache, sputum production, loss of smell and taste, hemoptysis, myalgia, fatigue, nausea, vomiting, diarrhoea, and abdominal pain. The ongoing epidemic of COVID-19 has spread very quickly, and currently, almost all countries have cases of COVID 19, totalling up 20,391.697 cases, and 743,724 deaths worldwide on December 8, 2020. Of the total cases 13,368.262 (65.6%) already have an outcome: 12,624.538 (94.4%) recovered and were discharged, but 743,724 people died (5.6%).^(1)^

Infection with SARS-Cov-2 can cause severe illness, and between 12% and 15% of all cases identified as positive for SARS-Cov-2 require admission to the intensive care unit (ICU).^(2)^

Although the percentage of COVID-19 patients that require ICU is not substantial, when this percentage is analysed as absolute numbers, the number of patients is sufficient to burden ICUs in the different health systems all over the world.^(3,4)^

Throughout this pandemic, much is said about the need for intensive care beds, mechanical ventilation devices, dialysis devices, and monitors.^(3-7)^ But for these physical resources to be effectively used, there is a need for extremely specialized human resources.

Since the beginning of this century, the World Health Organization (WHO) has warned about the increasing *deficit* of health workers.^(8)^ It is essential to point out that during a pandemic, human resources are highly susceptible to being infected and, therefore, the workplaces in ICUs are opened.^(9)^

## OBJECTIVE

To analyze the COVID-19 pandemic in Brazil, a continental-sized country, considered as an emerging economy but with several regional nuances, focusing on the availability of human resources, especially for intensive care units.

## METHODS

This study was conducted at the *Escola Paulista de Medicina* of *Universidade Federal de São Paulo*, São Paulo (SP), Brazil, in May and June 2020. To analyse the availability of human resources for ICUs, in the context of the COVID-19 pandemic in Brazil, we gathered data on health professionals from the database of the *Cadastro Nacional de Estabelecimentos de Saúde* (CNES).^(10)^ Next, we organized the professionals of interest delivering care for COVID-19 patients as follows: physicians, intensive care physicians, registered nurses, intensive care registered nurses, nurse technicians and intensive care nurse technicians, physical therapists, and respiratory therapists. Each of these professionals was then georeferenced, according to the city of their registration.

The number of professionals was initially correlated with the parameters, when available, used by the WHO Global Health Observatory Data Repository to compare different countries.^(11,12)^

The data for adult ICU beds in each federation unit (physical structure) were obtained from the Ministry of Health. In the sequence, we related them for each state, with the number of professionals for each ten ICU beds, as per the parameters of the Brazilian Intensive Care Medicine Association (AMIB - *Associação de Medicina Intensiva Brasileira*), which establish one intensivist, two registered nurses, five nurse technicians, and one respiratory therapist.^(13)^

The number of registered cases and deaths resulting from COVID-19 was also provided by the Ministry of Health and divided by states.

In the end, with the number of professionals, beds, and cases organized by locality, we identified in each state the care conditions for COVID-19, focusing mainly on ICUs.

With the current spread of the infection to the nonmetropolitan areas, we organized a scenario considering the human resources necessary to assist COVID-19 patients, excluding cases and professionals from the metropolitan regions.

## RESULTS

**Figure 1:**
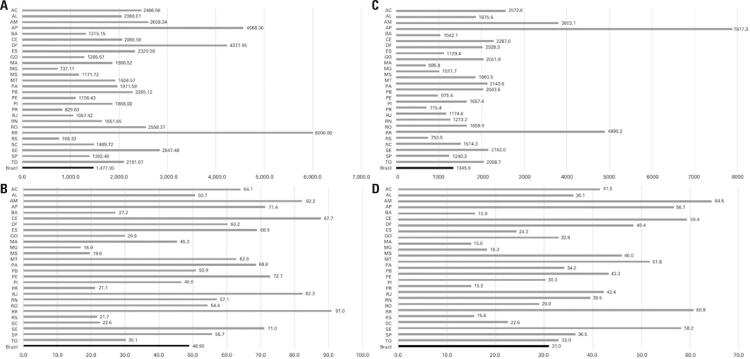
COVID-19 situation in Brazil until August 12, 2020. A) All cases; B) All deaths; C) Cases excluding the metropolitan regions; D) Deaths excluding the metropolitan regions

AC: Acre; AL: Alagoas; AM: Amazonas; AP: Amapá; BA: Bahia; CE: Ceará; DF: Distrito Federal; ES: Espírito Santo; GO: Goiás; MA: Maranhão; MG: Minas Gerais; MS: Mato Grosso do Sul; MT: Mato Grosso; PA: Paraíba; PE: Pernambuco; PI: Piauí; PR: Paraná; RJ: Rio de Janeiro; RN: Rio Grande do Norte; RO: Rondônia; RR: Roraima; RS: Rio Grande do Sul; SC: Santa Catarina; SE: Sergipe; SP: São Paulo; TO: Tocantins.

There were 3,109.630 (1,477.30 cases per 100 thousand inhabitants) cases of COVID-19 in Brazil, up to August 12, 2020, and the absolute number of deaths was 103,026 (48.90 deaths per 100 thousand inhabitants) (Figures 1A and 1B).

**Figure 2:**
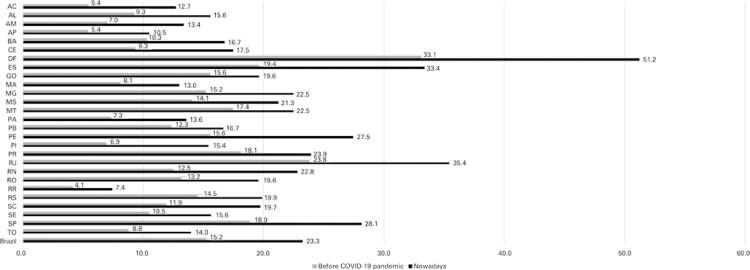
Adult intensive care unit beds per 100 thousand inhabitants in Brazil and its states

AC: Acre; AL: Alagoas; AM: Amazonas; AP: Amapá; BA: Bahia; CE: Ceará; DF: Distrito Federal; ES: Espírito Santo; GO: Goiás; MA: Maranhão; MG: Minas Gerais; MS: Mato Grosso do Sul; MT: Mato Grosso; PA: Paraíba; PE: Pernambuco; PI: Piauí; PR: Paraná; RJ: Rio de Janeiro; RN: Rio Grande do Norte; RO: Rondônia; RR: Roraima; RS: Rio Grande do Sul; SC: Santa Catarina; SE: Sergipe; SP: São Paulo; TO: Tocantins.

**Figure 3:**
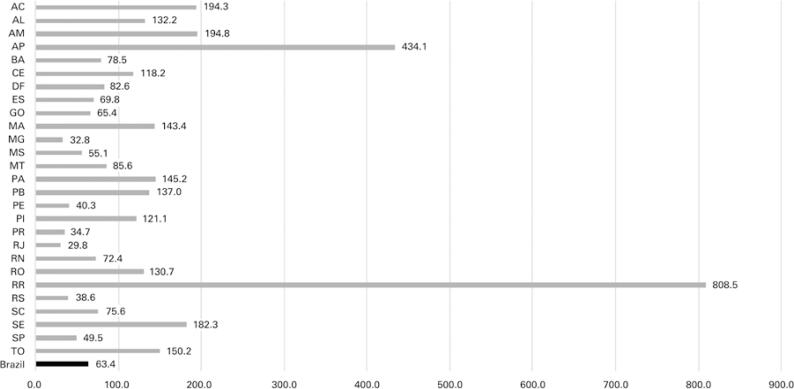
COVID-19 cases per intensive care unit beds in Brazil and its states

AC: Acre; AL: Alagoas; AM: Amazonas; AP: Amapá; BA: Bahia; CE: Ceará; DF: Distrito Federal; ES: Espírito Santo; GO: Goiás; MA: Maranhão; MG: Minas Gerais; MS: Mato Grosso do Sul; MT: Mato Grosso; PA: Paraíba; PE: Pernambuco; PI: Piauí; PR: Paraná; RJ: Rio de Janeiro; RN: Rio Grande do Norte; RO: Rondônia; RR: Roraima; RS: Rio Grande do Sul; SC: Santa Catarina; SE: Sergipe; SP: São Paulo; TO: Tocantins.

The total number of ICU beds for adults before the pandemic was 32,031 (15.24 beds per 100 thousand inhabitants). At the beginning of the pandemic, there was an expansion of the number of ICU beds. The Ministry of Health provided them along with the Health Secretariats of the states and cities, and the number of beds increased to 48,875 (23.26 beds per 100 thousand inhabitants) (Figure 2). Thus, there were 28.69 cases of COVID-19 per adult ICU beds in Brazil (Figure 3).

The total number per 100 thousand inhabitants of physicians, registered nurses, nurse technicians, and physical therapists in Brazil is, 196.84, 127.40, 245.10, and 39.71, respectively (Table 1). While the number of physicians was in accordance with the WHO recommendations, the number of nurses was not. Even if we gather registered nurses and nurse technicians (nursing staff), the figure would not be appropriate. When we correlated the number of specialized professionals, *i.e.*, intensivists, registered nurses and nurse technicians specialized in intensive care, besides respiratory therapists, required for every ten intensive care beds, we found a scenario in which, for any of these professional categories, there are not enough professionals. In Brazil, the health care providers to ten beds ratio were 0.634, 0.368, 0.621 and 0.100 for intensivists, ICU registered nurses, nurse technicians specialized in intensive care, and respiratory therapists, respectively (Table 1). Thus, only 10% of ICU beds in Brazil had a complete team of specialists to care for these patients (Table 2).

**Table 1:** Rates of healthcare professionals in Brazil and its states

State	Number of physicians per 100,000 inhabitants	Intensive care physicians per 10 ICU beds	Number of nurses per 100,000 inhabitants	Intensive care nurses per 10 ICU beds	Number of nurse technicians per 100,000 inhabitants	Intensive care nurse technicians per 10 ICU beds	Respiratory physical therapists per 10 ICU beds	Pulmonologists per 10 ICU beds	Infectious disease physicians per 10 ICU beds
Rondônia	142.4	0.546	108.1	0.029	252.0	1.063	0.057	0.230	0.862
Acre	108.4	0.089	118.9	0.089	249.1	NR	0.089	0.268	1.607
Amazonas	111.3	1.045	102.6	2.180	249.4	0.991	0.018	0.234	1.009
Roraima	136.2	0.667	129.1	NR	381.3	NR	NR	0.667	2.222
Pará	85.3	0.505	75.5	0.488	194.2	0.334	0.094	0.308	0.625
Amapá	95.3	0.337	109.6	NR	342.0	0.112	0.112	0.337	0.787
Tocantins	145.8	0.136	177.6	NR	381.0	0.182	0.045	0.227	0.500
Maranhão	80.9	0.369	106.1	0.554	225.5	0.543	0.011	0.261	0.239
Piauí	125.7	0.475	112.3	0.614	215.1	0.931	0.178	0.277	0.891
Ceará	125.9	0.595	114.6	0.269	147.2	0.044	0.044	0.294	0.589
Rio Grande do Norte	151.6	0.563	111.6	0.488	228.9	0.800	0.113	0.238	0.750
Paraíba	154.9	1.194	149.0	0.657	216.6	1.806	0.060	0.448	0.836
Pernambuco	157.2	0.675	117.4	0.511	241.2	0.335	0.293	0.229	0.423
Alagoas	131.6	0.346	100.9	0.288	159.4	0.942	0.038	0.442	0.519
Sergipe	162.3	0.251	101.7	0.084	157.6	0.111	0.056	0.334	0.891
Bahia	134.8	0.863	124.1	0.715	218.7	0.847	0.016	0.405	0.574
Minas Gerais	221.0	0.307	128.1	0.363	283.4	0.962	0.445	0.307	0.619
Espírito Santo	223.5	0.820	128.5	0.179	320.6	0.955	NR	0.395	0.679
Rio de Janeiro	248.6	0.918	139.8	0.294	265.5	0.451	0.059	0.459	0.680
São Paulo	260.0	0.671	143.1	0.183	226.0	0.632	0.048	0.372	0.947
Paraná	209.1	0.329	128.2	0.545	226.8	0.373	0.044	0.219	0.570
Santa Catarina	221.0	0.269	127.9	0.446	290.1	0.715	0.064	0.361	0.673
Rio Grande do Sul	243.6	0.565	138.2	0.552	353.2	1.064	0.031	0.658	0.702
Mato Grosso do Sul	195.4	0.491	127.8	0.203	249.3	0.321	0.017	0.203	0.677
Mato Grosso	148.3	0.549	123.0	0.294	270.1	0.447	0.064	0.217	0.600
Goiás	169.2	0.348	101.0	0.283	230.5	0.319	0.015	0.348	0.703
Distrito Federal	338.2	1.269	198.2	0.363	349.4	0.253	0.065	0.408	0.408
Brazil	196.8	0.634	127.4	0.368	245.1	0.621	0.100	0.360	0.711

ICU: intensive care unit; NR: not reported data.

**Table 2:** Number of complete teams of specialists in Brazil and its states to treat COVID-19 critically-ill patients

State	ICU beds assisted by intensive care physicians	ICU beds assisted by intensive care nurses	ICU beds assisted by intensive care nurse technicians	ICU beds assisted by respiratory therapists	Total number of ICU beds	Total number of complete ICU teams (human resources)	Complete teams per ICU beds (%)
Rondônia	190	5	74	20	348	5	1
Acre	10	5	0	10	112	0	0
Amazonas	580	605	110	10	555	10	2
Roraima	30	0	0	0	45	0	0
Pará	590	285	78	110	1,168	78	7
Amapá	30	0	2	10	89	0	0
Tocantins	30	0	8	10	220	0	0
Maranhão	340	255	100	10	921	10	1
Piauí	240	155	94	90	505	90	18
Ceará	950	215	14	70	1,596	14	1
Rio Grande do Norte	450	195	128	90	800	90	11
Paraíba	800	220	242	40	670	40	6
Pernambuco	1,770	670	176	770	2,624	176	7
Alagoas	180	75	98	20	520	20	4
Sergipe	90	15	8	20	359	8	2
Bahia	2,150	890	422	40	2,491	40	2
Minas Gerais	1,460	865	916	2,120	4,763	865	18
Espírito Santo	1,100	120	256	0	1,341	0	0
Rio de Janeiro	5,620	900	552	360	6,120	360	6
São Paulo	8,670	1,185	1,632	620	12,917	620	5
Paraná	900	745	204	120	2,736	120	4
Santa Catarina	380	315	202	90	1,412	90	6
Rio Grande do Sul	1,280	625	482	70	2,266	70	3
Mato Grosso do Sul	290	60	38	10	591	10	2
Mato Grosso	430	115	70	50	783	50	6
Goiás	480	195	88	20	1,379	20	1
Distrito Federal	1,960	280	78	100	1,544	78	5
Brazil	31,000	8,995	6,072	4,880	48,875	4,880	10

ICU: intensive care unit.

**Figure 4:**
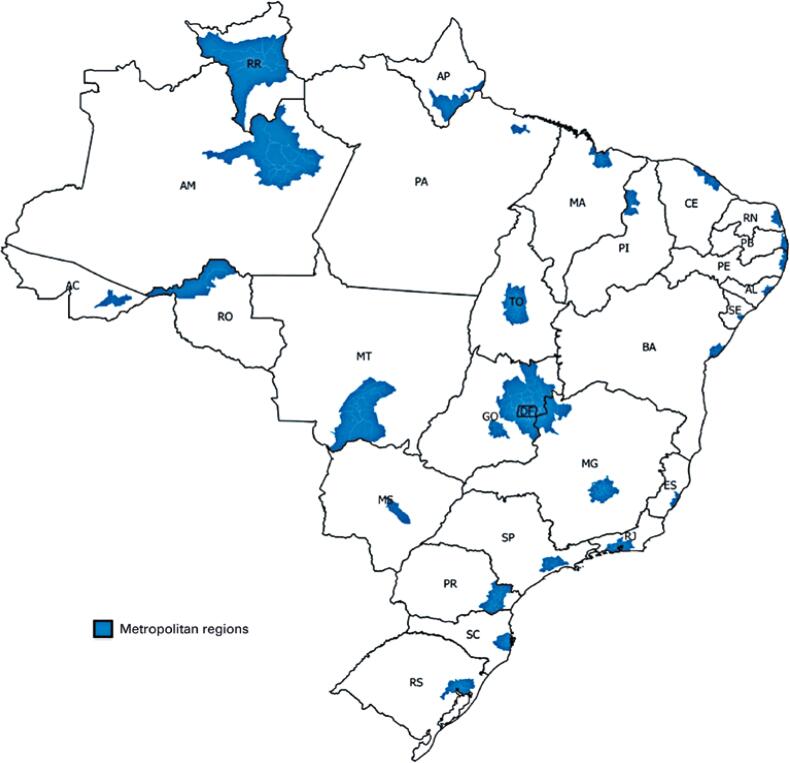
Map of Brazil showing the metropolitan region of each state

This situation got worse when we took into account the spreading of the infection to nonmetropolitan areas. When we excluded the metropolitan regions of the state capital cities, the nonmetropolitan areas of Brazil already had a higher number of cases than the metropolitan areas (52.5%), while during this research, the number of deaths was lower (36.5%) (Figure 4). We had 1,632.812 cases (1,345.88 cases per 100 thousand inhabitants) and 37,608 deaths (30.99 deaths per 100 thousand inhabitants) (Figures 1C and 1D).

**Figure 5:**
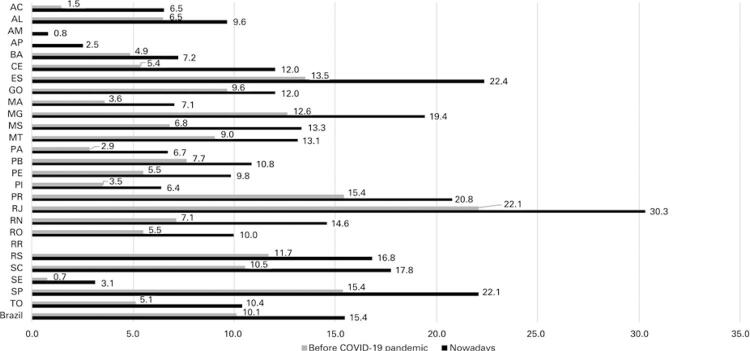
Adult intensive care unit beds per 100 thousand inhabitants in Brazil and its states, excluding metropolitan regions

AC: Acre; AL: Alagoas; AM: Amazonas; AP: Amapá; BA: Bahia; CE: Ceará; DF: Distrito Federal; ES: Espírito Santo; GO: Goiás; MA: Maranhão; MG: Minas Gerais; MS: Mato Grosso do Sul; MT: Mato Grosso; PA: Paraíba; PE: Pernambuco; PI: Piauí; PR: Paraná; RJ: Rio de Janeiro; RN: Rio Grande do Norte; RO: Rondônia; RR: Roraima; RS: Rio Grande do Sul; SC: Santa Catarina; SE: Sergipe; SP: São Paulo; TO: Tocantins.

**Figure 6:**
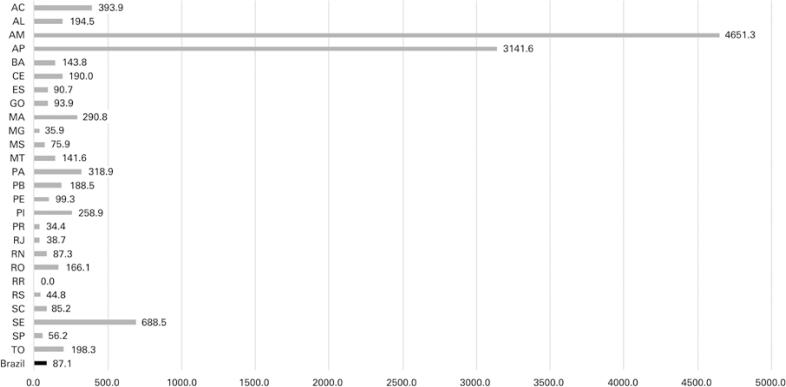
COVID-19 cases per intensive care unit beds in Brazil and its states, excluding the metropolitan regions

AC: Acre; AL: Alagoas; AM: Amazonas; AP: Amapá; BA: Bahia; CE: Ceará; DF: Distrito Federal; ES: Espírito Santo; GO: Goiás; MA: Maranhão; MG: Minas Gerais; MS: Mato Grosso do Sul; MT: Mato Grosso; PA: Paraíba; PE: Pernambuco; PI: Piauí; PR: Paraná; RJ: Rio de Janeiro; RN: Rio Grande do Norte; RO: Rondônia; RR: Roraima; RS: Rio Grande do Sul; SC: Santa Catarina; SE: Sergipe; SP: São Paulo; TO: Tocantins.

Before the pandemic, the nonmetropolitan areas of Brazil had 12,280 ICU beds for adults (10.12 beds for every 100 thousand inhabitants). After the availability of emergency ICU beds, there were 18,743 ICU beds for adults (15.45 beds for every 100 thousand inhabitants) (Figure 5). Thus, until August 12, 2020, we had 87.12 cases of COVID-19 for each ICU bed for adults in the nonmetropolitan areas of Brazil (Figure 6).

There was less availability of physicians, registered nurses, nurse technicians and physical therapists in the nonmetropolitan areas of Brazil, accounting for every 100 thousand inhabitants, 147.1, 111.5, 224.0 and 37.2, respectively (Table 3).

**Table 3:** Rate of healthcare specialists in Brazil and its states, excluding the metropolitan areas

State	Number of physicians per 100.000 inhabitants	Intensive care physicians per ten ICU beds	Number of nurses per 100.000 inhabitants	Intensive care nurses per ten ICU beds	Number of nurse technicians per 100.000 inhabitants	Intensive care nurse technicians per ten ICU beds	Respiratory physical therapists per ten ICU beds	Pulmonologists per ten ICU beds	Infectious disease physicians per ten ICU beds
Rondônia	108.8	0.410	89.7	NR	179.2	0.410	0.164	0.246	0.410
Acre	57.9	NR	89.8	NR	208.0	NR	NR	0.323	0.968
Amazonas	48.0	NR	86.9	NR	176.8	2.500	NR	NR	5.000
Roraima	58.0	NR	111.5	NR	330.7	NR	NR	NR	NR
Pará	47.3	0.196	65.1	0.342	176.4	0.489	0.024	0.049	0.440
Amapá	51.2	NR	85.8	NR	307.9	NR	NR	NR	NR
Tocantins	107.4	0.088	138.3	NR	325.5	0.354	NR	0.088	0.442
Maranhão	58.4	0.313	86.6	0.521	179.9	0.391	NR	0.156	0.234
Piauí	73.5	0.282	92.9	NR	148.3	0.775	NR	0.070	0.493
Ceará	74.3	0.132	96.9	0.066	133.8	0.017	0.033	0.149	0.182
Rio Grande do Norte	90.7	0.109	97.5	0.290	183.0	0.254	0.072	0.036	0.072
Paraíba	113.2	0.471	133.0	0.168	190.3	0.404	NR	0.404	0.539
Pernambuco	88.5	0.520	88.6	0.669	170.3	0.558	0.242	0.204	0.297
Alagoas	77.0	0.200	84.1	0.100	122.1	0.200	0.050	0.150	0.200
Sergipe	58.7	0.238	71.0	0.476	95.8	NR	NR	NR	0.238
Bahia	88.2	0.580	101.6	0.328	183.3	0.782	NR	0.404	0.454
Minas Gerais	172.1	0.243	117.8	0.236	253.0	0.858	0.181	0.291	0.465
Espírito Santo	162.4	0.373	105.7	0.000	244.5	0.132	NR	0.241	0.395
Rio de Janeiro	220.7	0.511	125.5	0.432	283.2	0.535	0.031	0.346	0.456
São Paulo	230.9	0.438	131.5	0.230	250.9	0.609	0.047	0.384	0.922
Paraná	165.1	0.136	129.2	0.842	215.4	0.458	0.068	0.149	0.532
Santa Catarina	200.7	0.132	122.0	0.406	282.0	0.775	0.038	0.340	0.539
Rio Grande do Sul	195.9	0.177	129.1	0.473	337.4	0.583	0.017	0.465	0.541
Mato Grosso do Sul	130.2	0.159	111.4	0.319	199.9	0.757	0.040	0.080	0.558
Mato Grosso	113.2	0.218	107.2	0.062	246.4	0.031	0.156	0.125	0.405
Goiás	119.5	0.256	94.0	0.085	217.7	0.455	0.028	0.199	0.540
Brazil	147.1	0.319	111.5	0.327	224.1	0.579	0.068	0.296	0.585

ICU: intensive care unit; NR: not reported data.

In nonmetropolitan areas of Brazil, for every ten ICU beds for adults, there was, respectively, 0.319 and 0.327, 0.579, and 0.068, intensivists and ICU registered nurses and nurse technicians specialized in intensive care, and respiratory therapists (Table 3). Only 7% of ICUs in the nonmetropolitan areas of Brazil had a complete ICU staff to care for patients (Table 4).

**Table 4:** Number of complete team of specialists in Brazil and its states to treat COVID-19 critically-ill patients, excluding the metropolitan areas

State	ICU beds assisted by intensive care physicians	ICU beds assisted by intensive care nurses	ICU beds assisted by intensive care nurse technicians	ICU beds assisted by respiratory therapists	Total number of ICU beds	Total number of complete ICU teams (human resources)	Complete teams per ICU beds (%)
Rondônia	50	0	10	20	122	0	0
Acre	0	0	0	0	31	0	0
Amazonas	0	0	6	0	12	0	0
Roraima	0	0	0	0	0	0	0
Pará	80	70	40	10	409	10	2
Amapá	0	0	0	0	5	0	0
Tocantins	10	0	8	0	113	0	0
Maranhão	120	100	30	0	384	0	0
Piauí	40	0	22	0	142	0	0
Ceará	80	20	2	20	605	2	0
Rio Grande do Norte	30	40	14	20	276	14	5
Paraíba	140	25	24	0	297	0	0
Pernambuco	280	180	60	130	538	60	11
Alagoas	40	10	8	10	200	8	4
Sergipe	10	10	0	0	42	0	0
Bahia	460	130	124	0	793	0	0
Minas Gerais	710	345	502	530	2,925	345	12
Espírito Santo	170	0	12	0	456	0	0
Rio de Janeiro	650	275	136	40	1,272	40	3
São Paulo	2,340	615	650	250	5,339	250	5
Paraná	220	680	148	110	1,616	110	7
Santa Catarina	140	215	164	40	1,058	40	4
Rio Grande do Sul	210	280	138	20	1,184	20	2
Mato Grosso do Sul	40	40	38	10	251	10	4
Mato Grosso	70	10	2	50	321	2	1
Goiás	90	15	32	10	352	10	3
Brazil	5,980	3,060	2,170	1,270	18,743	1,270	7

ICU: intensive care unit.

## DISCUSSION

Natural catastrophes, like floods and earthquakes, disasters like the bomb explosion in Beirut, or even bigger catastrophes produced by us, such as wars can sharply increase the number of severely-ill patients.^(14)^ Fortunately, these are uncommon situations. However, the beginning of a pandemic, such as the one we are facing with COVID-19, caused us to face a situation in which several patients develop respiratory or other organ dysfunction in a short time,^(15)^ and the major challenge of providing universal, timely, and affordable access to intensive care has become even more difficult.

Multiple adverse factors have emerged, such as some patients presenting with very severe disease, which we know little about it, and the consequent massive use of physical and human resources. Of especial concern is the work overload on the front line team, and the possibility of worsening this situation due to some team members getting infected and consequent absenteeism.^(16)^

However, most studies published only considered the number of hospital beds and equipment, relegating the availability of trained professionals to provide critical care much needed to critically ill patients with COVID-19 to a secondary level.^(2,4,6,7,17)^

Nevertheless, as we could demonstrate with this study, planning human resources is just as crucial as planning physical and structural resources.^(16)^The pandemic generated an urgent need for data on the number and qualification of health professionals that perform several tasks, and who have become crucial at this moment, especially to deliver care to severely-ill patients.

To the best of our knowledge, this is the first study that assesses the availability of human resources for the treatment of critically-ill COVID-19 patients. We analysed data from Brazil, a continental-sized country, with significant regional differences and a large population affected by COVID-19. However, we believe that this is not a problem inherent only to Brazil, but to most countries affected by the pandemic.^(18)^

The WHO has warned in recent years about the *deficit* of healthcare workers, and this may be a limiting factor to achieve the health-related 2030 Sustainable Development Goals.^(8,19)^ The WHO was unable to predict this epidemiological situation occurring ten years before the evaluation of the sustainable development goals.^(19)^

When considering the number of healthcare professionals, we demonstrated that the number of physicians and nursing professionals is in line with the WHO parameters. If we consider only registered nurses, we find that there are fewer of these professionals per 100 thousand inhabitants than the parameter suggested by the WHO. Nonetheless, when we evaluate the number of professionals outside the metropolitan areas, we find that the vast majority (13 out of 16) of states in the North and Northeast regions of Brazil, have fewer physicians than recommended by WHO. Concerning the nursing team, 18 out of 27 states do not have enough professionals. If we analyse only nurses, no state has enough number of these professionals.

The analysis considering professionals specialized in intensive care shows a very adverse situation. No state has enough professionals, of any category, whether in the metropolitan or nonmetropolitan areas. This finding was true to the number of beds that existed or, even worse, to the number of beds that were provided to meet the demand generated by the pandemic. According to our study, with the current number of professionals with declared qualifications, in the databases consulted, to work in ICUs, only 10% of total ICU beds would have a complete team. Thus, providing the appropriate number of professionals with proper training, especially for the vast nonmetropolitan areas of Brazil, is a great challenge. It is important to note that we use the parameter of the ratio of professionals per ten ICU beds provided by AMIB.^(13)^ However, ratio or even the level of care to be provided per patient, varies widely among different countries, because either there is staff shortage, or the policymakers do not agree there is an established need for professionals per bed.^(20)^

The quantity and specialization of the team are essential. As stated by Knaus et al., in the last century, in environments such as ICUs, the team’s specialization and the ability of these professionals to work in unison in the care of patients has direct impact on the outcome of patients.^(21)^ Although the use of invasive technologies, such as mechanical ventilation, renal replacement therapy, or even extracorporeal membrane oxygenation (ECMO) is crucial, they alone are not enough to provide the best care to critically-ill patients.^(20,21)^

Undoubtedly, the lack of ICU beds claimed many lives during this pandemic.^(3)^ The lack of access to the ICU beds puts patients at risk for delaying admission to the ICU and implementation of essential therapeutic measures, in addition to causing premature discharge and cancellation of invasive procedures.^(6)^ However, the availability of ICU beds without sufficient and adequately trained personnel also puts patients at risk.^(2)^

Among the limitations of this study, we must mention the data sources. There may be inaccuracies regarding the number of professionals specialized in intensive care, since it is almost certain that many of the physicians identified in the database as providers of intensive care are not intensivists, but rather hospitalists. On the other hand, the data used represent the professionals who are effectively working at the ICUs. Moreover, we did not assess the training of this workforce. There is also an inaccuracy (underreporting) in the number of cases and deaths by COVID-19, in Brazil.^(22)^

One should also note the calculations were carried out not taking into account the working and rest hours these specialized professionals must have, or already have, as provided by the law. Another factor that we did not consider was the number of ICU workers who were infected by SARS-COV2, and removed from duty shifts. Hence, the *deficit* of professionals for ICUs may be greater than we have demonstrated.

## CONCLUSION

There is a shortage of professionals for intensive care units, as we have shown in relation to Brazil. However, the expansion of intensive care is probably necessary to deal with some underlying problems of the various health systems, such as improper primary care, an aging population, and more complex and high-risk medical therapies, in addition to possible natural catastrophes, disasters, armed conflicts and outbreaks of infectious diseases. Therefore, governments and public policymakers, as well as hospital administrators, must be aware and organized to increase availability of intensive care unit beds. At the same time, they must pay attention not only to infrastructure and supplies, but also to intensive care unit professionals, in their training and the management of this essential resource. Otherwise, who will turn on the ventilators?
