# Supplementary material for: Who is going to turn on the ventilators?
Source: Einstein (Sao Paulo). 2021 Oct 13;19:eAO6211. doi: 10.31744/einstein_journal/2021AO6211 (PMC8522706; doi:10.31744/einstein_journal/2021AO6211)
Supplement: Translation

# Translation: Quem vai ligar os ventiladores?

**Authors:** Marcelo Cunio Machado Fonseca, Gabriela Tannus Branco de Araújo, Fulvio Alexandre Scorza, Paulo Sérgio Lucas da Silva, Teresa Raquel de Moraes Andrade, Daniela Farah, Dayan Sansone

**Affiliations:** 1 Brasil Escola Paulista de Medicina, Universidade Federal de São Paulo, São Paulo, SP, Brasil.; 2 Brasil Hospital do Servidor Público Municipal de São Paulo, São Paulo, SP, Brasil.

**Keywords:** COVID-19, Infecções por coronavírus, SARS-CoV-2, Pessoal de saúde, Unidades de terapia intensiva

## Abstract

**Objetivo:**

Analisar a pandemia da COVID-19 no Brasil, um país de dimensões continentais, considerado uma economia emergente, mas com inúmeras diferenças regionais, abordando a disponibilidade de recursos humanos, especialmente para unidades de terapia intensiva.

**Métodos:**

Foi acessado o banco de dados do Cadastro Nacional de Estabelecimentos de Saúde. Os profissionais de saúde que atuavam nos cuidados para COVID-19 foram georreferenciados. O número de profissionais foi correlacionado com os parâmetros utilizados pela Organização Mundial da Saúde. De acordo com a Associação de Medicina Intensiva Brasileira, correlacionaram-se os dados de leitos de terapia intensiva adulta em cada unidade federativa com o número de profissionais para cada dez leitos de terapia intensiva. Os números de profissionais, leitos e casos foram, então, organizados por unidade federativa.

**Resultados:**

O número de médicos por 100 mil habitantes seguiu as recomendações da Organização Mundial da Saúde; mas não o número de enfermeiras. O número de intensivistas, enfermeiros, técnicos de enfermagem especializados em terapia intensiva e fisioterapeutas respiratórios, necessário a cada dez leitos de terapia intensiva, não foi suficiente para nenhuma dessas categorias profissionais. Uma equipe completa desses especialistas esteve disponível para 10% dos leitos de terapia intensiva do Brasil.

**Conclusão:**

Há carência de profissionais para unidade de terapia intensiva, como demonstrado no Brasil. Os recursos físicos da terapia intensiva, para serem usados de forma eficiente, precisam de recursos humanos extremamente especializados; portanto, o planejamento de recursos humanos é tão crucial quanto o planejamento de recursos físicos e estruturais.

## INTRODUÇÃO

A doença pelo coronavírus 2019 (COVID-19) é uma doença respiratória viral causada pelo coronavírus da síndrome respiratória aguda grave 2 (SARS-CoV-2), que foi detectada pela primeira vez em dezembro de 2019, em Wuhan, na China. Os pacientes podem ser classificados como assintomáticos ou sintomáticos, e o quadro clínico inclui uma ampla variedade de sintomas inespecíficos, como febre, tosse seca, dispneia, cefaleia, produção de escarro, perda de olfato e paladar, hemoptise, mialgia, fadiga, náusea, vômitos, diarreia e dor abdominal. A epidemia de COVID-19 se espalhou de forma muito rápida, e, atualmente, quase todos os países do mundo têm casos de COVID-19, totalizando 20.391,697 casos e 743.724 mortes em todo o mundo, em 8 de dezembro de 2020. Do total de casos, 13.368,262 (65,6%) já tiveram um desfecho: 12.624,538 (94,4%) recuperaram-se e tiveram alta; porém, 743.724 pessoas morreram (5,6%).^(1)^

A infecção pelo SARS-CoV-2 pode causar quadro grave, e de 12% a 15% de todos os casos identificados como positivos para SARS-CoV-2 exigem internação em unidade de terapia intensiva (UTI).^(2)^

A percentagem de pacientes com COVID-19 com necessidade de internação em UTI não é muito grande, porém, quando analisados os números absolutos, o número de pacientes é suficiente para sobrecarregar as UTIs nos diferentes sistemas de saúde em todo o mundo.^(3,4)^

Na atual pandemia, ouve-se falar com frequência sobre a necessidade de leitos de terapia intensiva, dispositivos de ventilação mecânica e monitores.^(3-7)^ No entanto, para que esses recursos físicos possam ser usados com eficiência, também são necessários recursos humanos altamente especializados.

Desde o início deste século, a Organização Mundial da Saúde (OMS) tem alertado sobre um *déficit* crescente no número de profissionais de saúde.^(8)^ É importante ressaltar que, durante uma pandemia, os recursos humanos estão altamente suscetíveis a serem infectados, e existem, assim, vagas para profissionais de UTI.^(9)^

## OBJETIVO

Analisar a pandemia da COVID-19 no Brasil, um país de dimensões continentais, considerado uma economia emergente, mas com inúmeras diferenças regionais, abordando a disponibilidade de recursos humanos, especialmente para unidades de terapia intensiva.

## MÉTODOS

Este estudo foi realizado na Escola Paulista de Medicina da Universidade Federal de São Paulo, em São Paulo (SP), Brasil em maio e junho de 2020. Para analisar a disponibilidade de recursos humanos em UTIs, no contexto da pandemia da COVID-19 no Brasil, coletaram-se dados sobre profissionais da saúde no banco de dados do Cadastro Nacional de Estabelecimentos de Saúde (CNES).^(10)^ Depois, os profissionais de interesse no atendimento de pacientes com COVID-19 foram organizados da seguinte forma: médicos, médicos intensivistas, enfermeiros, enfermeiros intensivistas, técnicos de enfermagem, técnicos de enfermagem intensivistas, fisioterapeutas e fisioterapeutas respiratórios. Cada um desses grupos profissionais foi georreferenciado, de acordo com o respectivo município de registro.

O número de profissionais foi inicialmente correlacionado com os parâmetros, quando disponíveis, do *Global Health Observatory Data Repository* da OMS, para comparar os diferentes países.^(11,12)^

Os dados sobre leitos de UTI adulta em cada unidade federativa (estrutura física) foram obtidos do Ministério da Saúde. Na sequência, listaram-se os leitos para cada unidade federativa, juntamente do número de profissionais para cada dez leitos de terapia intensiva, conforme os parâmetros da Associação de Medicina Intensiva Brasileira (AMIB), que estabelecem um médico intensivista, dois enfermeiros, cinco técnicos de enfermagem e um fisioterapeuta respiratório.^(13)^

O número de casos registrados e mortes decorrentes da COVID-19 também foi disponibilizado pelo Ministério da Saúde e dividido por unidades federativas.

Por último, com o número de profissionais, leitos e casos organizados por localidade, identificaram-se, em cada unidade federativa, as condições de atendimento à COVID-19, com foco principal nas UTIs.

Com a atual disseminação da infecção para áreas não metropolitanas, organizou-se um cenário, tendo em vista os recursos humanos necessários para atender aos pacientes com COVID-19, excluindo-se os casos e profissionais de regiões metropolitanas.

## RESULTADOS

Foram registrados 3.109,630 (1.477,30 casos por 100 mil habitantes) casos de COVID-19 no Brasil até 12 de agosto de 2020, e o número absoluto de mortes foi 103.026 (48,90 mortes por 100 mil habitantes) (Figuras 1A e 1B).

**Figura 1:** Situação da COVID-19 no Brasil até 12 de agosto de 2020. A) Todos os casos; B) todas as mortes; C) casos excluindo as regiões metropolitanas; D) mortes excluindo as regiões metropolitanas

AC: Acre; AL: Alagoas; AM: Amazonas; AP: Amapá; BA: Bahia; CE: Ceará; DF: Distrito Federal; ES: Espírito Santo; GO: Goiás; MA: Maranhão; MG: Minas Gerais; MS: Mato Grosso do Sul; MT: Mato Grosso; PA: Paraíba; PE: Pernambuco; PI: Piauí; PR: Paraná; RJ: Rio de Janeiro; RN: Rio Grande do Norte; RO: Rondônia; RR: Roraima; RS: Rio Grande do Sul; SC: Santa Catarina; SE: Sergipe; SP: São Paulo; TO: Tocantins.

O número total de leitos de UTI adulta antes da pandemia era de 32.031 (15,24 leitos por 100 mil habitantes). No início da pandemia, houve uma expansão no número de leitos de UTI. O Ministério da Saúde providenciou os leitos, juntamente das Secretarias de Saúde dos estados e municípios, e o número aumentou para 48.875 (23,26 leitos por 100 mil habitantes) (Figura 2). Foram, assim, 28,69 casos de COVID-19 por leito de UTI adulta no Brasil (Figura 3).

**Figura 2:** Leitos de terapia intensiva adulta por 100 mil habitantes no Brasil e nas unidades federativas

AC: Acre; AL: Alagoas; AM: Amazonas; AP: Amapá; BA: Bahia; CE: Ceará; DF: Distrito Federal; ES: Espírito Santo; GO: Goiás; MA: Maranhão; MG: Minas Gerais; MS: Mato Grosso do Sul; MT: Mato Grosso; PA: Paraíba; PE: Pernambuco; PI: Piauí; PR: Paraná; RJ: Rio de Janeiro; RN: Rio Grande do Norte; RO: Rondônia; RR: Roraima; RS: Rio Grande do Sul; SC: Santa Catarina; SE: Sergipe; SP: São Paulo; TO: Tocantins.

**Figura 3:** Casos de COVID-19 por leito de terapia intensiva no Brasil e em suas unidades federativas

AC: Acre; AL: Alagoas; AM: Amazonas; AP: Amapá; BA: Bahia; CE: Ceará; DF: Distrito Federal; ES: Espírito Santo; GO: Goiás; MA: Maranhão; MG: Minas Gerais; MS: Mato Grosso do Sul; MT: Mato Grosso; PA: Paraíba; PE: Pernambuco; PI: Piauí; PR: Paraná; RJ: Rio de Janeiro; RN: Rio Grande do Norte; RO: Rondônia; RR: Roraima; RS: Rio Grande do Sul; SC: Santa Catarina; SE: Sergipe; SP: São Paulo; TO: Tocantins.

O número total por 100 mil habitantes de médicos, enfermeiros, técnicos de enfermagem e fisioterapeutas no Brasil foi, respectivamente, 196,84, 127,40, 245,10 e 39,71 (Tabela 1). O número de médicos atendeu à recomendação da OMS, mas não o de enfermeiros. Mesmo se fossem agrupados os enfermeiros e os técnicos de enfermagem (equipe de enfermagem), o valor ainda não seria suficiente. Quando correlacionado o número de profissionais especializados, ou seja, médicos, enfermeiros e técnicos de enfermagem intensivistas, além de fisioterapeutas respiratórios, necessário para cada dez leitos de terapia intensiva, chega-se a um cenário em que, para todas essas categorias, o número de profissionais não foi suficiente. No Brasil, o número de profissionais da saúde para cada dez leitos foi 0,634, 0,368, 0,621 e 0,100 para médicos intensivistas, enfermeiros intensivistas, técnicos de enfermagem intensivistas e fisioterapeutas respiratórios, respectivamente (Tabela 1). Somente 10% dos leitos de UTI no Brasil tinham uma equipe completa de especialistas para atendimento desses pacientes (Tabela 2).

**Tabela 1:** Taxa de profissionais de saúde no Brasil e nas unidades federativas

Unidade federativa	Número de médicos por 100 mil habitantes	Médicos intensivistas por 10 leitos de UTI	Número de enfermeiros por 100 mil habitantes	Enfermeiros intensivistas por 10 leitos de UTI	Número de técnicos de enfermagem por 100 mil habitantes	Técnicos de enfermagem intensivistas por 10 leitos de UTI	Fisioterapeutas respiratórios por 10 leitos de UTI	Pneumologistas por 10 leitos de UTI	Infectologistas por 10 leitos de UTI
Rondônia	142,4	0,546	108,1	0,029	252,0	1,063	0,057	0,230	0,862
Acre	108,4	0,089	118,9	0,089	249,1	NN	0,089	0,268	1,607
Amazonas	111,3	1,045	102,6	2,180	249,4	0,991	0,018	0,234	1,009
Roraima	136,2	0,667	129,1	NN	381,3	NN	NN	0,667	2,222
Pará	85,3	0,505	75,5	0,488	194,2	0,334	0,094	0,308	0,625
Amapá	95,3	0,337	109,6	NN	342,0	0,112	0,112	0,337	0,787
Tocantins	145,8	0,136	177,6	NN	381,0	0,182	0,045	0,227	0,500
Maranhão	80,9	0,369	106,1	0,554	225,5	0,543	0,011	0,261	0,239
Piauí	125,7	0,475	112,3	0,614	215,1	0,931	0,178	0,277	0,891
Ceará	125,9	0,595	114,6	0,269	147,2	0,044	0,044	0,294	0,589
Rio Grande do Norte	151,6	0,563	111,6	0,488	228,9	0,800	0,113	0,238	0,750
Paraíba	154,9	1,194	149,0	0,657	216,6	1,806	0,060	0,448	0,836
Pernambuco	157,2	0,675	117,4	0,511	241,2	0,335	0,293	0,229	0,423
Alagoas	131,6	0,346	100,9	0,288	159,4	0,942	0,038	0,442	0,519
Sergipe	162,3	0,251	101,7	0,084	157,6	0,111	0,056	0,334	0,891
Bahia	134,8	0,863	124,1	0,715	218,7	0,847	0,016	0,405	0,574
Minas Gerais	221,0	0,307	128,1	0,363	283,4	0,962	0,445	0,307	0,619
Espírito Santo	223,5	0,820	128,5	0,179	320,6	0,955	NN	0,395	0,679
Rio de Janeiro	248,6	0,918	139,8	0,294	265,5	0,451	0,059	0,459	0,680
São Paulo	260,0	0,671	143,1	0,183	226,0	0,632	0,048	0,372	0,947
Paraná	209,1	0,329	128,2	0,545	226,8	0,373	0,044	0,219	0,570
Santa Catarina	221,0	0,269	127,9	0,446	290,1	0,715	0,064	0,361	0,673
Rio Grande do Sul	243,6	0,565	138,2	0,552	353,2	1,064	0,031	0,658	0,702
Mato Grosso do Sul	195,4	0,491	127,8	0,203	249,3	0,321	0,017	0,203	0,677
Mato Grosso	148,3	0,549	123,0	0,294	270,1	0,447	0,064	0,217	0,600
Goiás	169,2	0,348	101,0	0,283	230,5	0,319	0,015	0,348	0,703
Distrito Federal	338,2	1,269	198,2	0,363	349,4	0,253	0,065	0,408	0,408
Brasil	196,8	0,634	127,4	0,368	245,1	0,621	0,100	0,360	0,711

UTI: unidade de terapia intensiva; NN: dados não notificados.

**Tabela 2:** Número de equipes completas de especialistas no Brasil e nas unidades federativas para tratar pacientes críticos com COVID-19

Unidade federativa	Leitos de UTI atendidos por médicos intensivistas	Leitos de UTI atendidos por enfermeiros intensivistas	Leitos de UTI atendidos por técnicos de enfermagem intensivistas	Leitos de UTI atendidos por fisioterapeutas respiratórios	Número total de leitos de UTI	Número total de equipes completas por leitos de UTI (recursos humanos)	Equipes completas por leitos de UTI (%)
Rondônia	190	5	74	20	348	5	1
Acre	10	5	0	10	112	0	0
Amazonas	580	605	110	10	555	10	2
Roraima	30	0	0	0	45	0	0
Pará	590	285	78	110	1.168	78	7
Amapá	30	0	2	10	89	0	0
Tocantins	30	0	8	10	220	0	0
Maranhão	340	255	100	10	921	10	1
Piauí	240	155	94	90	505	90	18
Ceará	950	215	14	70	1.596	14	1
Rio Grande do Norte	450	195	128	90	800	90	11
Paraíba	800	220	242	40	670	40	6
Pernambuco	1.770	670	176	770	2.624	176	7
Alagoas	180	75	98	20	520	20	4
Sergipe	90	15	8	20	359	8	2
Bahia	2.150	890	422	40	2.491	40	2
Minas Gerais	1.460	865	916	2.120	4.763	865	18
Espírito Santo	1.100	120	256	0	1.341	0	0
Rio de Janeiro	5.620	900	552	360	6.120	360	6
São Paulo	8.670	1.185	1.632	620	12.917	620	5
Paraná	900	745	204	120	2.736	120	4
Santa Catarina	380	315	202	90	1.412	90	6
Rio Grande do Sul	1.280	625	482	70	2.266	70	3
Mato Grosso do Sul	290	60	38	10	591	10	2
Mato Grosso	430	115	70	50	783	50	6
Goiás	480	195	88	20	1.379	20	1
Distrito Federal	1.960	280	78	100	1.544	78	5
Brasil	31.000	8.995	6.072	4.880	48.875	4.880	10

UTI: unidade de terapia intensiva.

Essa situação ficou ainda pior quando foi levada em conta a disseminação da infecção para áreas não metropolitanas. Quando excluídas as regiões metropolitanas das capitais estaduais, as áreas não metropolitanas do Brasil já tinham um número mais alto de casos que as áreas metropolitanas (52,5%), embora, até o momento desta pesquisa, o número de mortes fosse mais baixo (36,5%) (Figura 4). Foram registrados 1.632,812 casos (1.345,88 casos por 100 mil habitantes) e 37.608 mortes (30,99 mortes por 100 mil habitantes) (Figuras 1C e 1D).

**Figura 4:** Mapa do Brasil mostrando a região metropolitana de cada unidade federativa

Antes da pandemia, as áreas não metropolitanas do Brasil tinham 12.280 leitos de UTI para adultos (10,12 leitos por 100 mil habitantes). Após a disponibilização de leitos de UTI emergenciais, existiam 18.743 leitos de UTI para adultos (15,45 leitos por 100 mil habitantes) (Figura 5). Assim, até 12 de agosto de 2020, foram registrados 87,12 casos de COVID-19 para cada leito de UTI adulta nas áreas não metropolitanas do Brasil (Figura 6).

**Figura 5:** Leitos de unidades de terapia intensiva para adultos por 100 mil habitantes no Brasil e nas unidades federativas, excluindo as regiões metropolitanas

AC: Acre; AL: Alagoas; AM: Amazonas; AP: Amapá; BA: Bahia; CE: Ceará; DF: Distrito Federal; ES: Espírito Santo; GO: Goiás; MA: Maranhão; MG: Minas Gerais; MS: Mato Grosso do Sul; MT: Mato Grosso; PA: Paraíba; PE: Pernambuco; PI: Piauí; PR: Paraná; RJ: Rio de Janeiro; RN: Rio Grande do Norte; RO: Rondônia; RR: Roraima; RS: Rio Grande do Sul; SC: Santa Catarina; SE: Sergipe; SP: São Paulo; TO: Tocantins.

**Figura 6:** Casos de COVID-19 por leito de terapia intensiva no Brasil e unidades federativas, excluindo-se as regiões metropolitanas

AC: Acre; AL: Alagoas; AM: Amazonas; AP: Amapá; BA: Bahia; CE: Ceará; DF: Distrito Federal; ES: Espírito Santo; GO: Goiás; MA: Maranhão; MG: Minas Gerais; MS: Mato Grosso do Sul; MT: Mato Grosso; PA: Paraíba; PE: Pernambuco; PI: Piauí; PR: Paraná; RJ: Rio de Janeiro; RN: Rio Grande do Norte; RO: Rondônia; RR: Roraima; RS: Rio Grande do Sul; SC: Santa Catarina; SE: Sergipe; SP: São Paulo; TO: Tocantins.

Foi menor a disponibilidade de médicos, enfermeiros, técnicos de enfermagem e fisioterapeutas nas áreas não metropolitanas do Brasil, sendo, para cada 100 mil habitantes, 147,1, 111,5, 224,0 e 37,2, respectivamente (Tabela 3).

**Tabela 3:** Taxa de profissionais de saúde no Brasil e nas unidades federativas, excluindo as regiões metropolitanas

Unidade federativa	Número de médicos por 100 mil habitantes	Médicos intensivistas por 10 leitos de UTI	Número de enfermeiros por 100 mil habitantes	Enfermeiros intensivistas por 10 leitos de UTI	Número de técnicos de enfermagem por 100 mil habitantes	Técnicos de enfermagem intensivistas por 10 leitos de UTI	Fisioterapeutas respiratórios por 10 leitos de UTI	Pneumologistas por 10 leitos de UTI	Infectologistas por 10 leitos de UTI
Rondônia	108,8	0,410	89,7	NN	179,2	0,410	0,164	0,246	0,410
Acre	57,9	NN	89,8	NN	208,0	NN	NN	0,323	0,968
Amazonas	48,0	NN	86,9	NN	176,8	2,500	NN	NN	5,000
Roraima	58,0	NN	111,5	NN	330,7	NN	NN	NN	NN
Pará	47,3	0,196	65,1	0,342	176,4	0,489	0,024	0,049	0,440
Amapá	51,2	NN	85,8	NN	307,9	NN	NN	NN	NN
Tocantins	107,4	0,088	138,3	NN	325,5	0,354	NN	0,088	0,442
Maranhão	58,4	0,313	86,6	0,521	179,9	0,391	NN	0,156	0,234
Piauí	73,5	0,282	92,9	NN	148,3	0,775	NN	0,070	0,493
Ceará	74,3	0,132	96,9	0,066	133,8	0,017	0,033	0,149	0,182
Rio Grande do Norte	90,7	0,109	97,5	0,290	183,0	0,254	0,072	0,036	0,072
Paraíba	113,2	0,471	133,0	0,168	190,3	0,404	NN	0,404	0,539
Pernambuco	88,5	0,520	88,6	0,669	170,3	0,558	0,242	0,204	0,297
Alagoas	77,0	0,200	84,1	0,100	122,1	0,200	0,050	0,150	0,200
Sergipe	58,7	0,238	71,0	0,476	95,8	NN	NN	NN	0,238
Bahia	88,2	0,580	101,6	0,328	183,3	0,782	NN	0,404	0,454
Minas Gerais	172,1	0,243	117,8	0,236	253,0	0,858	0,181	0,291	0,465
Espírito Santo	162,4	0,373	105,7	0,000	244,5	0,132	NN	0,241	0,395
Rio de Janeiro	220,7	0,511	125,5	0,432	283,2	0,535	0,031	0,346	0,456
São Paulo	230,9	0,438	131,5	0,230	250,9	0,609	0,047	0,384	0,922
Paraná	165,1	0,136	129,2	0,842	215,4	0,458	0,068	0,149	0,532
Santa Catarina	200,7	0,132	122,0	0,406	282,0	0,775	0,038	0,340	0,539
Rio Grande do Sul	195,9	0,177	129,1	0,473	337,4	0,583	0,017	0,465	0,541
Mato Grosso do Sul	130,2	0,159	111,4	0,319	199,9	0,757	0,040	0,080	0,558
Mato Grosso	113,2	0,218	107,2	0,062	246,4	0,031	0,156	0,125	0,405
Goiás	119,5	0,256	94,0	0,085	217,7	0,455	0,028	0,199	0,540
Brasil	147,1	0,319	111,5	0,327	224,1	0,579	0,068	0,296	0,585

UTI: unidade de terapia intensiva; NN: dados não notificados.

Nas áreas não metropolitanas do Brasil, para cada dez leitos de UTI para adultos, existiam, respectivamente, 0,319, 0,327, 0,579 e 0,068 médicos intensivistas, enfermeiros, técnicos de enfermagem intensivistas e fisioterapeutas respiratórios (Tabela 3). Somente 7% das UTIs nas áreas não metropolitanas do Brasil tinham uma equipe de UTI completa para atendimento aos pacientes (Tabela 4).

**Tabela 4:** Número de equipes completas de especialistas no Brasil e nas unidades federativas para tratar pacientes críticos com COVID-19, excluindo as áreas metropolitanas

Unidade federativa	Leitos de UTI atendidos por médicos intensivistas	Leitos de UTI atendidos por enfermeiros intensivistas	Leitos de UTI atendidos por técnicos de enfermagem intensivistas	Leitos de UTI atendidos por fisioterapeutas respiratórios	Número total de leitos de UTI	Número total de equipes completas por leitos de UTI (recursos humanos)	Equipes completas por leitos de UTI (%)
Rondônia	50	0	10	20	122	0	0
Acre	0	0	0	0	31	0	0
Amazonas	0	0	6	0	12	0	0
Roraima	0	0	0	0	0	0	0
Pará	80	70	40	10	409	10	2
Amapá	0	0	0	0	5	0	0
Tocantins	10	0	8	0	113	0	0
Maranhão	120	100	30	0	384	0	0
Piauí	40	0	22	0	142	0	0
Ceará	80	20	2	20	605	2	0
Rio Grande do Norte	30	40	14	20	276	14	5
Paraíba	140	25	24	0	297	0	0
Pernambuco	280	180	60	130	538	60	11
Alagoas	40	10	8	10	200	8	4
Sergipe	10	10	0	0	42	0	0
Bahia	460	130	124	0	793	0	0
Minas Gerais	710	345	502	530	2.925	345	12
Espírito Santo	170	0	12	0	456	0	0
Rio de Janeiro	650	275	136	40	1.272	40	3
São Paulo	2.340	615	650	250	5.339	250	5
Paraná	220	680	148	110	1.616	110	7
Santa Catarina	140	215	164	40	1.058	40	4
Rio Grande do Sul	210	280	138	20	1.184	20	2
Mato Grosso do Sul	40	40	38	10	251	10	4
Mato Grosso	70	10	2	50	321	2	1
Goiás	90	15	32	10	352	10	3
Brasil	5.980	3.060	2.170	1.270	18.743	1.270	7

UTI: unidade de terapia intensiva.

## DISCUSSÃO

Catástrofes naturais, como enchentes e terremotos, desastres, como a explosão de uma bomba em Beirute, ou catástrofes ainda maiores produzidas pelo homem, como guerras, podem aumentar bruscamente o número de pacientes críticos.^(14)^ Felizmente, essas situações são raras. No entanto, com o surgimento de uma pandemia, como a da COVID-19, encontramo-nos em uma situação em que muitos pacientes desenvolvem disfunção respiratória ou de outros órgãos em um curto período de tempo.^(15)^ O grande desafio de garantir acesso a cuidados intensivos de maneira universal e oportuna e com valor que se pode pagar torna-se ainda mais difícil.

Múltiplos fatores adversos já surgiram, como alguns pacientes acometidos pela doença muito grave, sobre a qual pouco sabemos, e a enorme utilização de recursos físicos e humanos decorrentes dessa situação. É importante ressaltar a sobrecarga de trabalho da equipe de primeira linha e a possibilidade de agravamento dessa situação, devido ao contágio dos membros da equipe e ao consequente absenteísmo.^(16)^

No entanto, a maioria dos estudos publicados considerou apenas o número de leitos hospitalares e equipamentos, relegando a um nível secundário a disponibilidade de profissionais capacitados a prestar cuidados intensivos extremamente necessários aos pacientes críticos de COVID-19.^(2,4,6,7,17)^

Porém, como demonstrado neste estudo, o planejamento de recursos humanos é tão essencial quando o planejamento de recursos físicos e estruturais.^(16)^A pandemia gerou uma necessidade urgente de dados sobre o número e a qualificação dos profissionais de saúde que realizam diversas funções e estão se tornando essenciais neste momento, especialmente para o atendimento de pacientes críticos.

Até onde se sabe, este é o primeiro estudo que avalia a disponibilidade de recursos humanos para o tratamento de pacientes críticos de COVID-19. Foram analisados dados do Brasil, um país continental, com diferenças regionais consideráveis e uma grande população afetada pela COVID-19. No entanto, acredita-se que este não é um problema inerente apenas ao Brasil, mas comum à maioria dos países afetados pela pandemia.^(18)^

A OMS alertou recentemente sobre o *déficit* de trabalhadores da saúde, e isso pode ser um fator limitante para que se cumpram as metas de saúde dos Objetivos de Desenvolvimento Sustentável 2030.^(8,19)^ A OMS não tinha como prever que essa situação epidemiológica ocorreria 10 anos antes da avaliação de tais metas.^(19)^

Quando analisado o número de profissionais de saúde, demonstra-se que o número de médicos e de profissionais de enfermagem atende aos parâmetros da OMS. Se considerados apenas os enfermeiros, há menos desses profissionais por 100 mil habitantes do que estabelecem os parâmetros da OMS. Contudo, quando avaliado o número de profissionais fora das áreas metropolitanas, a maioria (13 dos 16) das unidades federativas nas regiões Norte e Nordeste do Brasil tem menos médicos que o recomendado pela OMS. Em relação à equipe de enfermagem, 18 das 27 unidades federativas não têm profissionais suficientes. Se apenas os enfermeiros forem considerados, descobre-se que nenhuma unidade federativa tem número suficiente desses profissionais.

A análise que considera profissionais especializados em terapia intensiva mostra uma situação bastante adversa. Nenhuma unidade federativa tem profissionais suficientes, de nenhuma categoria, seja em áreas metropolitanas ou não metropolitanas. Isso era verdadeiro para o número de leitos que já existiam e agravou-se ainda mais para os leitos fornecidos para atender à demanda gerada pela pandemia. Segundo este estudo, com o número atual de profissionais com qualificações declaradas, nos bancos de dados consultados, para trabalhar em UTI, somente 10% dos leitos totais de UTI teriam uma equipe completa. Assim, oferecer um número apropriado de profissionais, com a capacitação correta, principalmente para as vastas áreas não metropolitanas do Brasil, é um grande desafio. É importante notar que é usada como parâmetro a razão de profissionais para cada dez leitos de UTI, conforme informado pela AMIB.^(13)^ Porém, a razão, ou mesmo o nível de cuidados prestados por paciente, varia muito entre os diferentes países, seja porque existe carência de profissionais, ou porque os formuladores de políticas públicas não concordam que exista uma necessidade estabelecida de um certo número de profissionais por leito.^(20)^

A quantidade e a especialização da equipe são essenciais. Segundo Knaus et al., no último século, em ambientes como as UTIs, a especialização da equipe e a habilidade desses profissionais de trabalhar de forma conjunta nos cuidados têm impacto direto no desfecho dos pacientes.^(21)^ Embora o uso de tecnologias invasivas, como ventilação mecânica, terapia renal substitutiva, ou mesmo oxigenação por membrana extracorpórea (ECMO), seja crucial, essas medidas sozinhas não garantem o melhor atendimento de pacientes críticos.^(20,21)^

Certamente, a falta de leitos de UTI ceifou muitas vidas durante essa pandemia.^(3)^ A falta de acesso a leitos de UTI coloca em risco os pacientes devido à demora na internação e para implementação de medidas terapêuticas essenciais, além de resultar em altas prematuras e cancelamento de procedimentos invasivos.^(6)^ No entanto, a disponibilidade de leitos de UTI sem profissionais suficientes e adequadamente capacitados também coloca em risco os pacientes.^(2)^

Entre as limitações deste estudo, é preciso mencionar as próprias fontes de dados. Pode haver imprecisão em relação ao número de profissionais especializados em terapia intensiva, já que é quase certo que muitos dos médicos identificados no banco de dados como prestadores de cuidados intensivos talvez não sejam intensivistas, mas, sim, hospitalistas. Por outro lado, os dados usados representam os profissionais que efetivamente trabalham nas UTIs. Além disso, não foi avaliada a capacitação dessa mão de obra. Há também imprecisão (subnotificação) no número de casos e mortes por COVID-19, no Brasil.^(22)^

Também deve-se observar que os cálculos foram realizados sem considerar as horas de trabalho e de repouso de que esses profissionais especializados devem dispor, ou já dispõem, de acordo com a legislação. Outro fator que não se considerou foi o número de trabalhadores de UTI infectados pelo SARS-CoV-2 e que, por isso, foram removidos de seus turnos. Portanto, o *déficit* de profissionais nas UTIs pode ser ainda maior que o demonstrado.

## CONCLUSÃO

Há uma carência de profissionais em unidades de terapia intensiva, conforme demonstrado para o Brasil. No entanto, a expansão da terapia intensiva é provavelmente necessária para o enfrentamento de alguns problemas de base encontrados nos vários sistemas de saúde, como Atenção Primária insatisfatória, envelhecimento da população e terapias cada vez mais complexas e de mais alto risco, além de possíveis catástrofes naturais, desastres, conflitos armados e surtos de doenças infecciosas. Desse modo, os governos e os formuladores de políticas públicas, bem com os administradores hospitalares, devem estar cientes e se organizar para aumentar a disponibilidade de leitos de terapia intensiva. Ao mesmo tempo, devem estar atentos não apenas à infraestrutura e aos insumos, mas também aos profissionais de unidades de terapia intensiva, bem como à capacitação e à gestão desses recursos essenciais. Caso contrário, quem vai ligar os ventiladores?
